# Effectiveness of interventions for changing HIV related risk behaviours among key populations in low-income setting: A Meta-Analysis, 2001–2016

**DOI:** 10.1038/s41598-020-58767-0

**Published:** 2020-02-10

**Authors:** Keshab Deuba, Diksha Sapkota, Upendra Shrestha, Rachana Shrestha, Bir Bahadur Rawal, Komal Badal, Kathleen Baird, Anna Mia Ekström

**Affiliations:** 1 National Centre for AIDS and STD Control/Global Fund Programs, Kathmandu, Nepal; 2 Save the Children Country Office Nepal, Kathmandu, Nepal; 3 School of Nursing and Midwifery, Gold Coast University Hospital, Griffith University, Gold Coast, Australia; 4 Public Health and Environment Research Center, Lalitpur, Nepal; 5 National Centre for AIDS and STD Control, Ministry of Health and Population, Kathmandu, Nepal; 6 UNAIDS, Kathmandu, Nepal; 7 Department of Global Public Health, Karolinska Institutet, Stockholm, Sweden; 8 Department of Infectious Diseases, Karolinska University Hospital, Stockholm, Sweden

**Keywords:** Epidemiology, Outcomes research

## Abstract

The aim of this review was to conduct a meta-analysis to assess the effectiveness of behavioural interventions to reduce HIV-related risk behaviours among key populations: people who inject drugs, female sex workers, men who have sex with men and transgender in Nepal over the last two decades. Using four electronic databases, we performed a systematic search of the literature on HIV interventions implemented in Nepal and published from January 2001 to December 2016. In addition, grey literature was also scrutinised for potential articles. The search focussed specifically on behavioural interventions (peer education and HIV testing services) targeted for key populations. Random-effects models were used to calculate the pooled odds ratio for dichotomous outcomes (condom use in last sex or unsafe injection practices), pooled HIV prevalence and subgroup analyses by age groups and epidemic zones in Nepal. Forty-three studies with 15,642 participants were included (people who inject drugs: 7105; men who have sex with men and transgender: 2637; female sex workers: 5900). Pooled prevalence showed a higher occurrence of HIV among people who inject drugs (12%) followed by men who have sex with men/transgender (5%) and female sex workers (2%) respectively. There was a significant increase in the odds of condom use among female sex workers, men who have sex with men and transgender who received peer education interventions in both informal and formal setting compared to those who did not. Similarly, the odds of condom use among female sex workers, men who have sex with men and transgender improved significantly among those who received HIV counselling and testing services as compared to those who did not use such services. Subgroup analyses also verified the effectiveness of these interventions for both young and adult key populations and across all three epidemic zones. However, none of the included interventions were found to be effective for reducing unsafe injection practices among people who inject drugs. HIV prevention interventions in Nepal have effectively reduced risky behaviours among female sex workers, men who have sex with men and transgender over the last two decades but not among people who inject drugs. This calls for continued implementation of existing efforts as well as for new interventions adapted to the needs of people who inject drugs.

## Introduction

The world has committed to ending the HIV epidemic by 2030. Accomplishments such as a decrease in AIDS-related mortality by 45% since its peak in 2005 and increase in people receiving antiretroviral therapy (ART) by one-third in just two years have inspired global confidence that this target is achievable^1^. Several behavioural, biomedical and structural interventions operating at an individual and population/community level are being widely used for HIV prevention and support^2,3^.

The HIV epidemic in Nepal is concentrated, with nearly 60% of infections occurring in key populations. Among the key populations excluding male labour migrants, men who have sex with men (MSM) and transgender (TG) accounts for 9% of total infections followed by 6% among male sex workers (MSWs), 6% among clients of female sex workers (FSW), 4% among people who inject drugs (PWID), and 1% among FSW^4^. In 2013, UNAIDS proposed to move from the strategy ‘know your epidemic’ to ‘know your local epidemics’ emphasizing the need to address specific issues within local epidemics to improve the HIV response^5^. HIV prevalence is not uniform across Nepal, but rather associated with the extension of highways, mobility trends and urbanization. Based on these epidemic characteristics, four epidemic zones (Kathmandu Valley, Pokhara Valley, Terai Highway and West to Far west Hills) have been identified in Nepal^5^. Nearly, 50% of the total number of HIV infections are recorded along the highway districts across the country which share an open border with India, and 31% in Kathmandu valley^6^. In Pokhara valley, one of the major tourist destinations of Nepal, the HIV epidemic is driven by the FSW and PWID, whereas in West to Far west Hills, the burden of HIV is highest among seasonal male labour migrants to India. Secondary analysis of HIV prevalence surveys identified diverging trends in risk behaviours across local epidemic zones, which suggested the need for context-specific HIV prevention policies and activities^7^.

In Nepal, 16,913 people living with HIV were estimated to be receiving ART from 74 sites as of December 2018. Notwithstanding this, it is estimated that 37% of those linked to HIV care are yet to initiate ART^8^. As of July 2018, there were over 175 HIV counselling and testing services (HTS) including 136 government sites in Nepal, but 33% of people living with HIV were still unaware of their HIV status^8,9^. In Asia, 95% of all new HIV infections occur in young key populations. This is also true in the Nepalese context, where greater proportions of sex workers and PWID were less than 25 years of age, and relative lack of knowledge regarding HIV infection among them (72% of young females and 56% of young males) as well as limited negotiation power in terms of safer sex, makes them particularly vulnerable to HIV acquisition^10^. Young people often face stigma and discrimination in the society where they are living, which further marginalise them and limit their access to HIV services. Young people are also less likely to identify themselves as drug users or sex workers, making them particularly hard to reach, less responsive to communication and less willing to use services or adopt protective behaviours^2,11^.

Several reviews have examined impact of HIV-related interventions using behavioural outcomes^12–15^; however, they have been concentrated in one type of key population only. Two reviews included studies conducted among MSM only^12,13^ and another review had included studies involving FSW only^15^. Furthermore, most of the reviews have included peer-driven interventions only and do not include grey literature^12–14^. In addition, there is a substantial heterogeneity in results across studies. A review of Chinese studies, for example, reported the effectiveness of interventions on reducing HIV risk behaviours, but not the prevalance of HIV and syphilis^12^. Similarly, another review reported improvement in HIV testing only as a result of peer-involvement interventions^13^. HIV prevention is mainly a sociocultural issue and interventions that have been successful and effective in one setting may not be effective in another setting. The capacity to respond to the HIV epidemic also differs from country to country, depending on the population affected and resources available^14^. This review aimed to address this gap by conducting a review of all available studies on behavioural interventions targeting at-risk populations for HIV in the context of Nepal. This review includes peer-driven interventions targeting key populations and HIV testing services, which are most commonly used in Nepal^16^.

Though the literature suggests the need of implementation of context-specific as well as age-specific programs for reduction and prevention of HIV, there are no such targeted interventions and blanket policy may not address varying needs and demands of different groups. However, developing targeted interventions could sometimes be too costly for low-resource settings such as Nepal. A lack of country-based information on the impact and quality of prevention programs poses challenges in development, implementation, and evaluation of HIV prevention programs. Hence, this meta-analysis aimed to assess the effectiveness of available HIV-related behavioural interventions for key populations in Nepal. In addition, the pooled prevalence of HIV was estimated, including any differences in HIV risk behaviours by age and local epidemic zone.

## Methods

**Figure 1:**
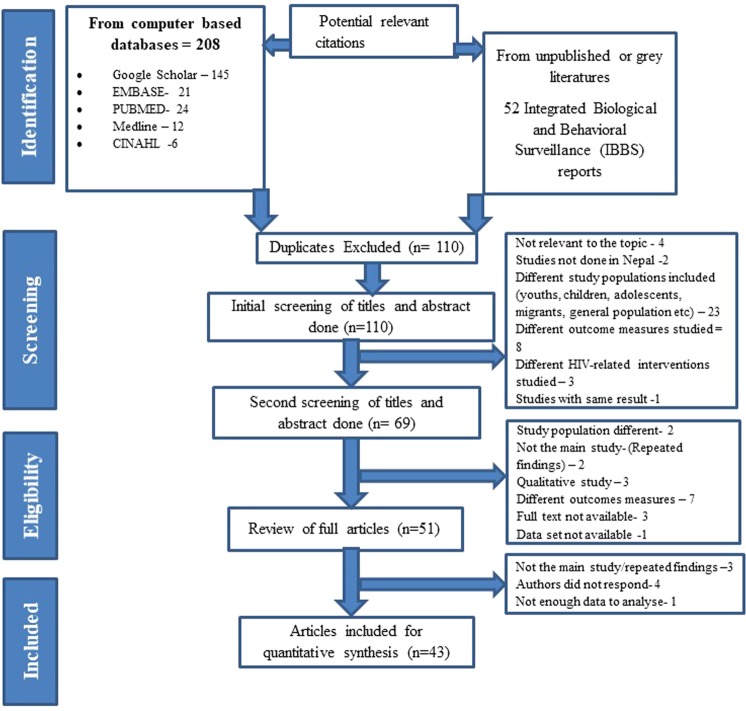
Flowchart using the PRISMA statement.

The Preferred Reporting Items for Systematic Reviews and Meta-analysis (PRISMA)^17^ statement was used to guide this review (Fig. 1).

### Inclusion criteria

Studies were included in the review if they met the following criteria:

- HIV-specific behavioural intervention, either peer-driven education and/or HTS was implemented in Nepal.
- Laboratory tests performed to assess the HIV incidence or prevalence among key populations (PWID, MSM, TG and FSW).
- HIV-related risk behaviours (condom use, unsafe needle/syringe use) were assessed.
- Studies conducted between January 2001- December 2016.
- If more than one article presented data for the same project and target population, only the study reporting above-mentioned outcomes was included.

### Data sources (search strategy)

For identification of relevant literature, a specific search strategy was employed using explicit inclusion criteria to avoid selection bias. A search was done between February 10^th^–28^th^ 2017 in four different databases: PubMed, Google Scholar, Excerpta Medica database (EMBASE) and Cumulative Index to Nursing and Allied Health Literature (CINAHL). Medical Subject Headings (MeSH) terms and keywords were used, and Boolean operators were applied for selecting the articles. A complete list of search terms are included in Supplementary Table S1. The references of the eligible articles were also searched, and the process was iterated until no new references were identified. Grey literature pertaining to the topic area such as reports, surveys, and policy documents prepared by national and international, government and non-government organizations was also reviewed. Websites of agencies involved in HIV-prevention, such as the United Nations Program on HIV and AIDS (UNAIDS), FHI360 and Population Council and conference abstracts (through Gateway and National Library of Medicine) were also searched for documents relates to HIV in Nepal.

### Types of interventions

Key behavioral interventions specifically aimed to prevent HIV and AIDS targeting PWID, TG, MSM and FSW are peer-driven interventions and HTS, which are based on the elements of several behavioral theories (social learning theory, theory of reasoned action, diffusion of innovation theory, theory of participatory education, and health belief model)^18,19^. These behavioural interventions have brought about several opportunities for behaviour modification: reducing the number of sexual partners; improving treatment adherence among people living with HIV; increasing use of clean needles among PWID; increasing correct and consistent use of condoms; and eventually, reducing the morbidity and mortality due to HIV and AIDS.

Peer-driven interventions include all the education sessions that are held in formal settings (drop-in centres) as well as informal settings (public cruising places such as parks, bus stops, restaurants, and temples). Peer educators and outreach educators are mostly selected from key populations and involved in disseminating HIV-related knowledge. They act as a bridge between key population and service providers, and distribute condoms, lubricants and new syringes. HTS is another essential intervention in HIV prevention and care, which includes client-oriented or provider-oriented counselling and testing services. It is an entry point to HIV treatment, care and support, including ART, and promotes HIV prevention and maintains linkages with key populations^20^.

Peer education and HTS are hypothesized to influence different intermediate outcomes^18,19^, such as increasing knowledge on HIV risk reduction strategies, beliefs, attitudes and behaviors, which in turn are believed to promote clients to take up safer and healthier behaviors. Components and causal pathways of behavioural interventions against HIV infection are depicted in Supplementary Fig. S1.

### Measures of exposure to different types of intervention

Standard indicators for monitoring the HIV testing and prevention programmes for key populations were included. The indicators include the percentage of key populations who received HIV testing in the last 12 months and who know the results and also the percentage of key populations reached by at least one of the prevention programs (outreach and peer education and/or HTS)^21^. These data were disaggregated by type of key population and age (16–24 years vs ≥24 years). Studies assessed self-reported participation in two-targeted behavioural interventions was assessed by the following questions:

i. Have you met or interacted with peer educators or outreach educators or community mobilizers or community educators in the last 12 months? (Reasons for meeting or interaction: discussion on how HIV or AIDS/Sexually Transmitted Infections (STI) is/is not transmitted, demonstration on using condom correctly, regular/non-regular use of condoms, and counselling on reducing number of sex partners).
ii. Have you visited or been to any drop-in centre in the last 12 months? (Reasons for visiting: to get STI treatment, to collect condoms, to learn the correct way of using a condom, to watch film on HIV and/or AIDS, to participate in discussions on HIV transmission, to participate in discussion on STI transmission, to participate in training, interaction and discussion programs on HIV, AIDS and STI).
iii. Have you visited any HTS centres in the last 12 months? (Reasons for visiting pre-post HIV test counselling, counselling on the correct use of condom, information on HIV window period, HIV test result and treatment, care and support services). In addition, discussions on safe injecting behaviour was also the reason among PWID for meeting peer educators, visiting a drop-in centre/HTS centre.

Self-reported HIV risk behaviours were also considered. For example: the proportion of FSW reporting condom use (yes/no) at last sexual encounter with a client; the proportion of MSM and TG reporting condom use (yes/no) at last time anal sex with a male partner; and the proportion of PWID who, in the past one week, had used needles or syringes (yes/no) that had been used by others before^21^. This meta-analysis included studies reporting at least one intermediary behavioural outcome/measures related to HIV prevention.

### Study selection

Initial inclusion/exclusion of studies was based on the title and abstract review done by two co-authors (KD and DS). Three authors (KD, DS and US) independently screened studies multiple times against the aforementioned inclusion criteria. Iterative discussions were conducted in case of discrepancies to reach consensus. Final inclusion/exclusion of studies was based on a thorough reading of the full-text article.

### Data extraction

For all eligible studies, the following information was extracted: first author, publication year, study setting, study design, sample sizes, population characteristics, interventions type, outcome of interest, and key findings. All the included studies were cross-sectional in nature (Supplementary Table S2).

### Methodological appraisal of the included studies

It was initially planned to use the Appraisal Tool for Cross-Sectional Studies (AXIS), consisting of 20 items, for quality appraisal of studies^22^. AXIS consists of 20 questions and has been developed to assess the quality and risk of bias of cross-sectional studies^22^. The study team discussed the relevance of conducting an appraisal as almost all the studies have used similar study designs (cross-sectional), methodology, analytical approaches, and outcomes. After discussions, it was concluded not to do methodological appraisal as it was assumed that it would give the same ratings for all studies. In addition, considering the limitations of the included cross-sectional studies, the study team decided to include all the studies.

### Meta-analytic methods

A logistic model was used to explore possible associations between participation in specific behavioural interventions and self-reported HIV risk behaviours. The standardized effect size was estimated using the odds ratio (OR) as all studies reported dichotomous outcomes and compared two groups (exposed and not exposed). Standard meta-analytic methods were used to derive standardized effect size estimates by directly entering the OR calculated from each study into Comprehensive Meta-AnalysisV.2.2. ORs were pooled using random effects model. For pooled HIV prevalence, statistics were shown using the event rate and 95% confidence interval (CI) were calculated within the Comprehensive Meta-Analysis software using the sample size (n) and standard error. Q test and I^2^ statistics were used to assess the statistical heterogeneity among studies. The choice of random effects model was based on conceptual grounds, regardless of the test of heterogeneity (I^2^ index and Q statistic), because this study aims to estimate the mean of the distribution of effects rather than one true effect^23^.

## Results

### Characteristics of study and interventions

Out of 260 potentially relevant articles identified through initial search, 110 remained after removing duplicates. After several rounds of screening, 43 articles were found to meet the pre-specified inclusion criteria. Amongst them, 42 included the integrated biological and behavioural surveillance surveys, which are part of the National HIV Surveillance Plan. These 42 studies were descriptive surveys using cross-sectional designs to monitor HIV/STI prevalence and assess behavioural information from key populations in Nepal. To ensure homogeneity to exposure to behavioural interventions, two studies among FSW of Kathmandu and Pokhara in 2004 were excluded from the meta-analysis due to lack of information regarding exposure to peer-driven interventions. However, these two studies were taken into account while calculating the pooled HIV prevalence. One study conducted among the FSW in the 22 Terai highway districts in 2004, was excluded due to unavailability of data related to outcome of interest^24^. Similarly, one study from 2004 among MSM and TG was not included in the assessment of effect size of interventions but included in estimating pooled HIV prevalence. A total of 22 studies involving PWID (males only) were included in the analysis of pooled HIV prevalence, but six of these were excluded when estimating effect size (see Supplementary Table S2). One study which involved female injecting drug users in the Kathmandu Valley was not included in meta-analysis as there was only one assessment without any follow-up^25^. Of the 43 studies, 16 were conducted in the Kathmandu Valley (the capital city of Nepal), 11 were conducted in the Pokhara Valley (one of the major tourist destinations of Nepal), 11 were conducted in Terai highway districts and 5 were conducted in west to far west Terai highway districts. Amongst the included studies, 42 were nationally representative surveys using a cross-sectional design, and one was a cross-sectional study conducted among MSM and TG in Kathmandu Valley (Supplementary Table S2).

The results are categorised by type of intervention and key populations targeted. For facilitate the interpretation of the results, the effectiveness of the interventions has been disaggregated by age and epidemic zone, i.e. Kathmandu Valley, Pokhara Valley and Terai highway districts.

### Pooled HIV prevalence

A pooled HIV prevalence of 5.0% (95% CI 3.8–6.7%) was calculated using data from the six studies conducted among MSM and TG (five in Kathmandu Valley and one in Terai highway district) (Table 1). Among FSW, a pooled HIV prevalence rate of 1.7% (95% CI 1.4–2.1%). was derived from 14 studies conducted between 2004 and 2016 across three different epidemic zones in Nepal. For PWID, we found a pooled HIV prevalence of 11.9%, (95% CI 8.3–16.7%), based on 22 studies conducted in three epidemic zones across the whole country between 2003 and 2015 (Table 1).

**Table 1:** Pooled HIV prevalence among key populations.

Key populations	Number of studies	HIV prevalence (%)	95% CI
PWID	22	11.9	8.3–16.7
MSM and TG	6	5.0	3.8–6.7
FSW	14	1.7	1.4–2.1

Abbreviations: CI, confidence interval.

We also analysed the trends in HIV prevalence using the Spectrum and AIDS Epidemic Model, estimating the number of people living with HIV in Nepal over time using different programmatic and surveillance survey inputs over the period of January 1990 to 2018 (Supplementary Fig. S2) and found very similar results compared to our pooled estimates indicating that the epidemic indeed has been stable between 2004 and 2016.

### Effectiveness of behavioural interventions for FSW

**Figure 2:**
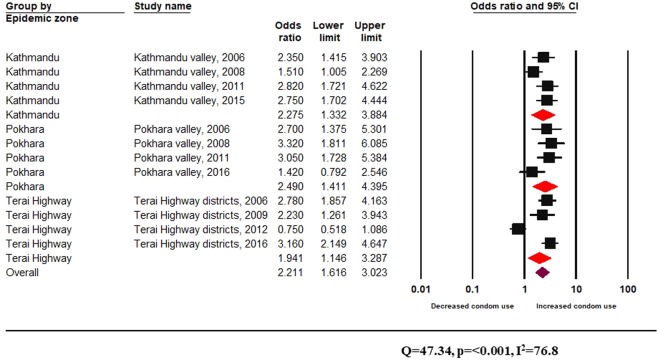
Effectiveness of peer-driven interventions (informal setting) across epidemic zones among FSW.

Twelve integrated biological and behavioural surveillance surveys had reported complete information regarding the effect on HIV risk behaviours following participation in peer-driven interventions in both informal and formal settings (Fig. 2).

Figure 2 shows statistically significant results of heterogeneity testing using Q value and an *I*^2^ value of 76.8%, indicating substantial heterogeneity across studies. The pooled effect size from these surveys shows a statistically significant impact of peer education on increased odds of condom use among FSW (random-effects pooled effect size OR 2.2, 95% CI 1.6–3.0) (Fig. 2). Stratifying the discrete random effect size estimates by epidemic zone, peer education in informal associated with increased condom use across all epidemic zones (Fig. 2).

**Figure 3:**
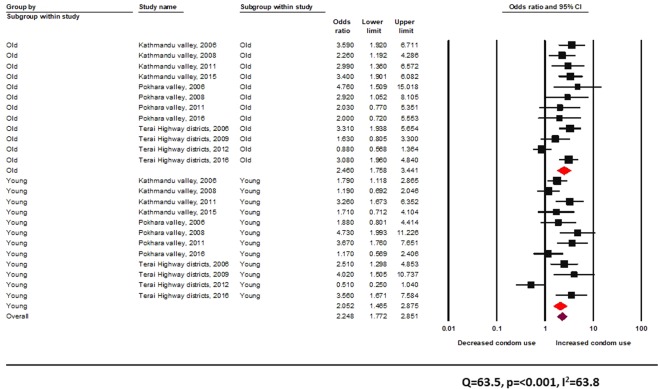
Effectiveness of peer-driven interventions (informal setting) across age groups among FSW.

The Q-value of 63.5 is statistically significant, and the *I*^2^ value indicates substantial heterogeneity across studies (Fig. 3). Peer-driven interventions in informal settings seemed effective in improving the odds of condom use among both young people (random effects pooled OR 2.0, 95% CI: 1.5–2.9) and adult participants (random effects pooled OR 2.5, 95% CI: 1.8–3.4) (Fig. 3).

**Figure 4:**
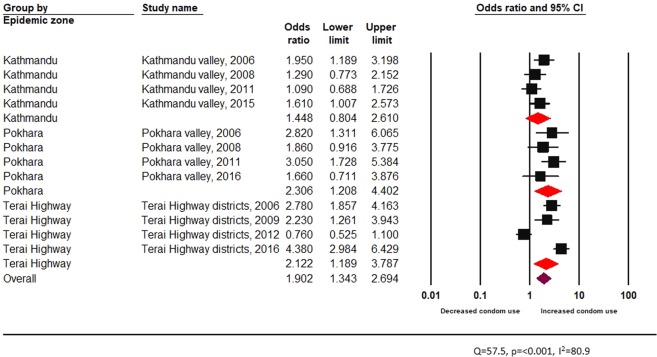
Effectiveness of peer driven interventions (formal setting) across epidemic zones among FSW.

Overall, peer education in informal setting is associated with a statistically significant increment in condom use among FSW (pooled OR 1.90, 95% CI 1.34–2.69) (Fig. 4). However, when compared across epidemic zones, peer-education in formal settings was not found to be effective in increasing the odds of condom use among FSW of Kathmandu Valley (pooled OR 1.45 95% CI 0.80–2.61) (Fig. 4).

**Figure 5:**
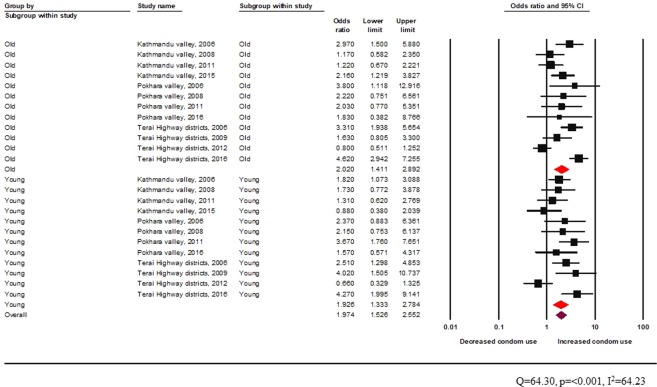
Effectiveness of peer driven interventions (formal setting) across age groups among FSW.

Age-wise comparison shows peer education provided from a formal setting to be effective in increasing condom use for both young and adult FSW (young: random effects pooled OR 1.9, 95% CI: 1.3–2.8, vs adult: random effects pooled OR 2.0, 95% CI: 1.4–2.9) (Fig. 5).

**Figure 6:**
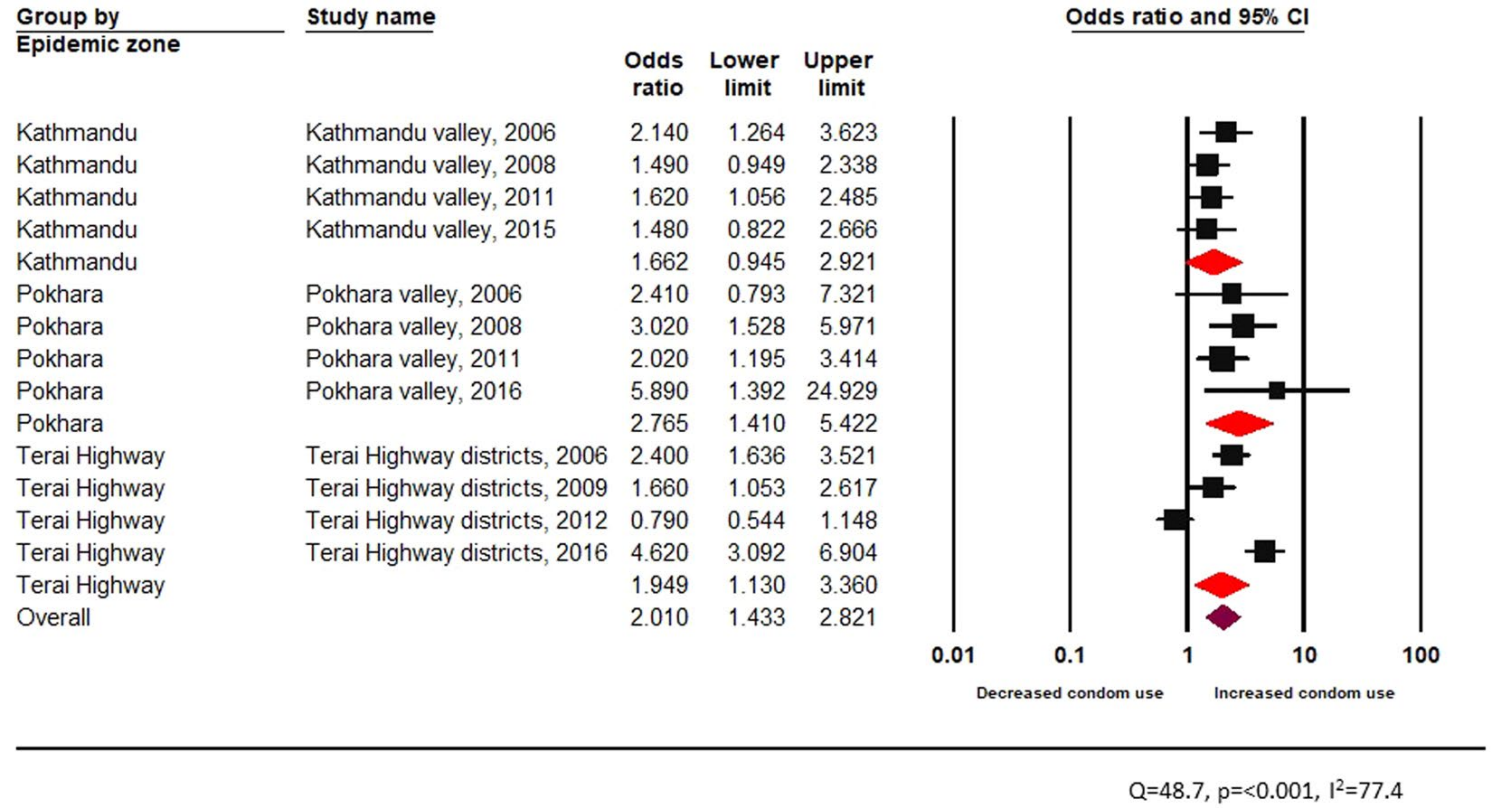
Effectiveness of HTS visit among FSW across epidemic zones.

In Fig. 6, a statistically significant Q statistic (p < 0.001) and a *I*^2^ value of 77.4 indicated substantial heterogeneity. Pooled estimates also indicated that HTS services were effective in terms of increasing condom use among FSW in all epidemic zones (pooled OR 2.0, 95% CI 1.4–2.8) (Fig. 6).

**Figure 7:**
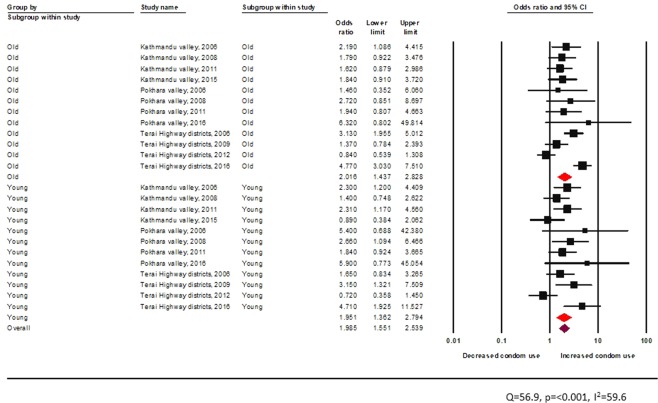
Effectiveness of HTS visit among female sex workers across age groups.

In Fig. 7, the Q statistic and *I*^2^ value showed substantial heterogeneity. Stratifying the discrete random effect size estimates by age groups, a visit to HTS was significantly associated with increased condom use across all age groups (young: random effects pooled OR 1.9, 95% CI: 1.4–2.8, vs adult: random effects pooled OR 2.0, 95% CI: 1.4–2.8) (Fig. 7).

### Effectiveness of behavioural interventions for MSM and TG

In total, seven studies were included and the effectiveness of practiced interventions was assessed only according to the age groups. Analysis by epidemic zones was not possible as five studies were from Kathmandu Valley and only one was done in Terai highway districts and Kathmandu Valley.

**Figure 8:**
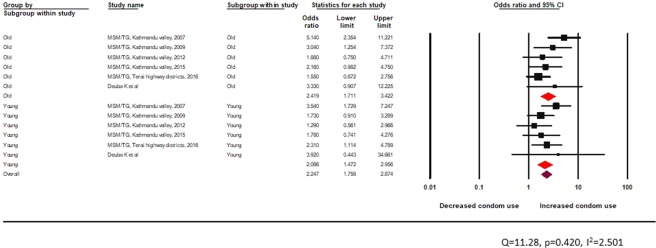
Effectiveness of peer-led intervention (informal setting) among MSM and TG across age groups.

The Q-value was not statistically significant, and the I^2^ value indicated almost no heterogeneity between studies. Overall, there was increased condom use among MSM and TG exposed to peer education in an informal setting (OR 2.3, 95% CI: 1.8–2.9). The peer-led intervention delivered through an informal setting was found to be effective in both age groups as there was increased odds of condom use among MSM and TG who met or interacted with peer educators in the previous 12 months (young: random effects pooled OR 2.1 95% CI 1.5–2.9 and adult: random effects pooled OR 2.4 95% CI 1.7–3.4) (Fig. 8).

**Figure 9:**
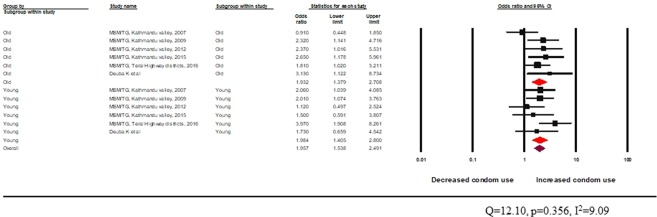
Effectiveness of peer-led interventions (formal setting) for MSM and TG across age groups.

The test of heterogeneity was not statistically significant. Peer-driven interventions in formal settings appeared effective in improving the odds of condom use behaviour among both age groups (young: random effects pooled OR 1.9, 95% CI: 1.4–2.8 vs adult: random effects pooled OR 1.9, 95% CI: 1.4–2.7) (Fig. 9).

**Figure 10:**
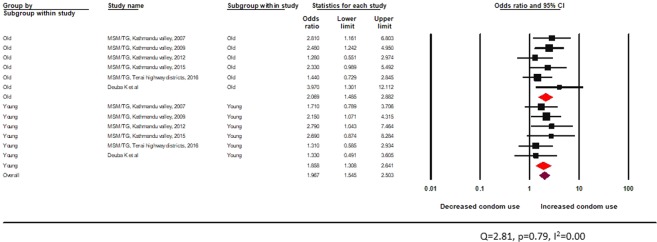
Effectiveness of HTS among MSM and TG across age groups.

The test of heterogeneity was not statistically significant (Fig. 10). HTS interventions including voluntary counseling and testing was found to significantly improve condom use behaviors among both age groups (young: random effects pooled OR 1.9, 95% CI: 1.3–2.6, vs adult: random effects pooled OR 2.1, 95% CI: 1.5–2.9) (Fig. 10).

### Effectiveness of behavioural interventions for PWID

Sixteen studies assessed the effectiveness of behavioural interventions on reducing unsafe injection during the study period, but we could not find any statistically significant peer-driven intervention and HTS aimed at reducing unsafe injections among PWID across the four epidemic zones of Nepal.

**Figure 11:**
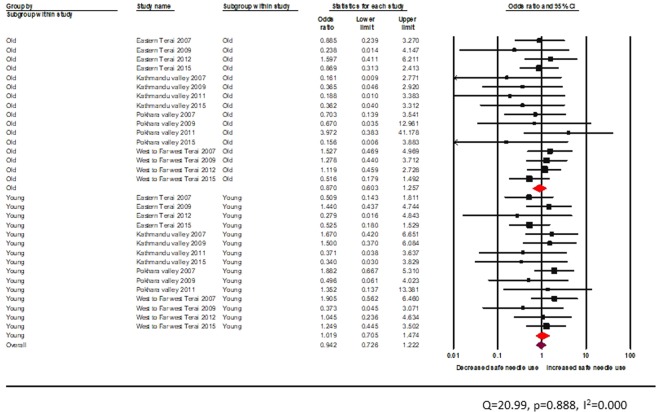
Effectiveness of peer-led intervention (informal setting) among PWID across age groups.

**Figure 12:**
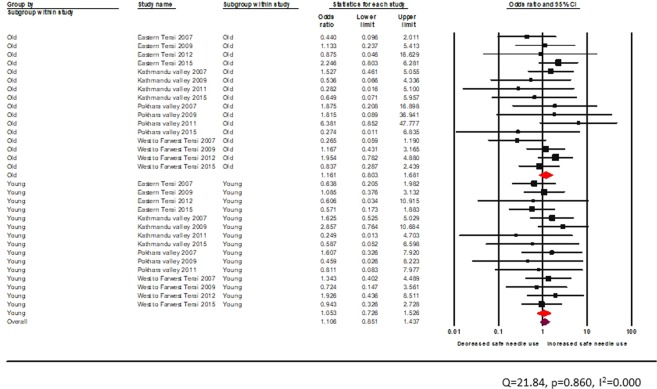
Effectiveness of peer-led intervention (formal setting) among PWID across age groups.

The test of heterogeneity was not statistically significant (Figs. 11 and 12). Age-wise comparison showed that peer education provided from informal settings was not effective in reducing unsafe injection for neither among young nor adult PWID (young: random effects pooled OR 1.0, 95% CI: 0.7–1.5, vs adult: random effects pooled OR 0.9, 95% CI: 0.6–1.3) (Fig. 11).

The test of heterogeneity was not statistically significant. There was no significant reduction in unsafe injection among young and adult PWID exposed to peer education in a formal setting (young: random effects pooled OR 1.1, 95% CI: 0.7–1.5, vs adult: random effects pooled OR 1.2, 95% CI: 0.8–1.7) (Fig. 12).

**Figure 13:**
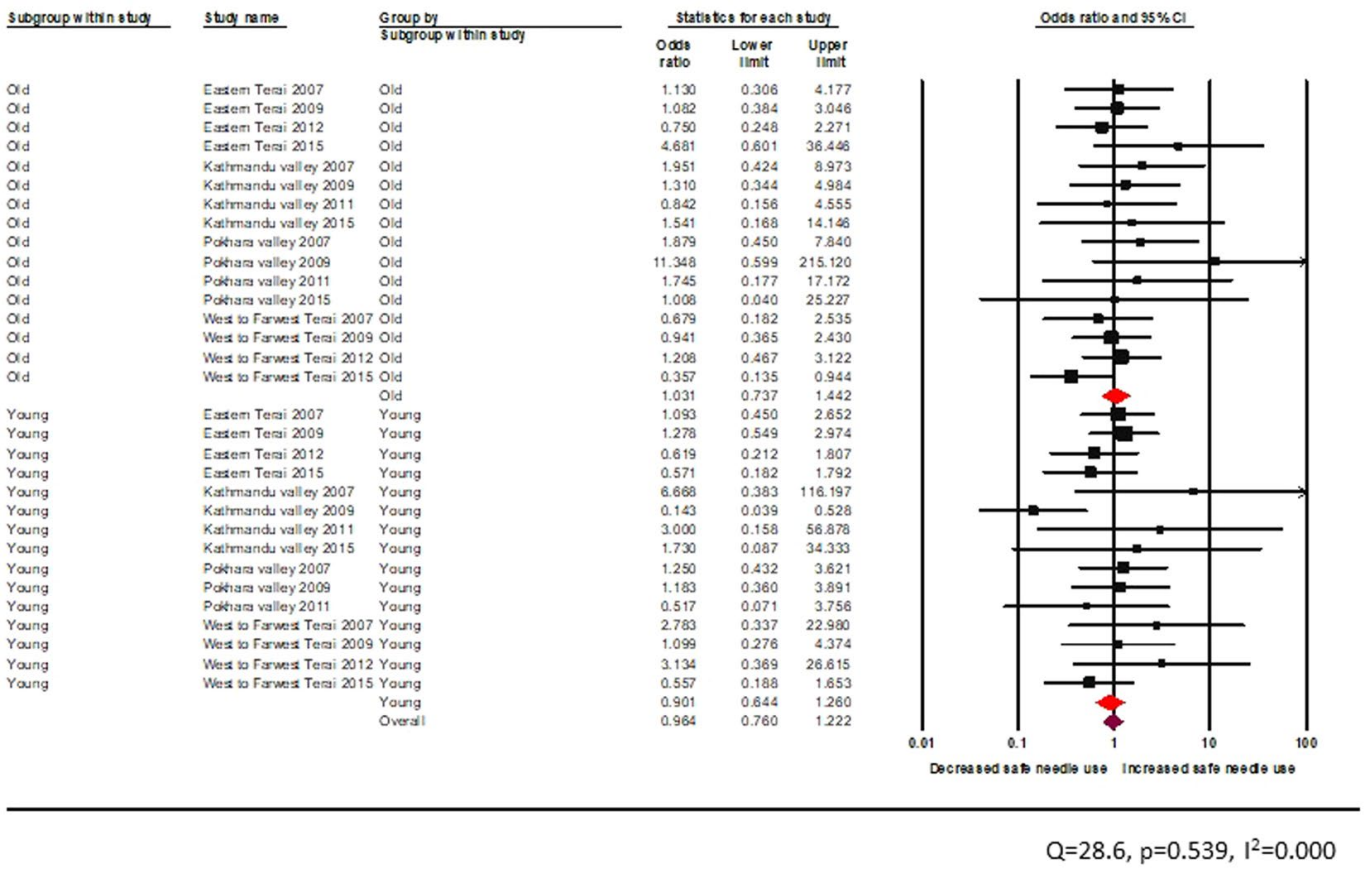
Effectiveness of HTS among PWID across age groups.

The test of heterogeneity was not statistically significant. A similar finding was obtained for PWID visiting the HTS facility for HIV counselling and testing (young: random effects pooled OR 0.90, 95% CI: 0.62–1.31, vs adult: random effects pooled OR 1.0, 95% CI: 0.7–1.4)) (Fig. 13).

## Discussion

To the best of our knowledge, this is the first meta-analysis of the effect of behavioural interventions on HIV risk behaviours among key populations (FSW, PWID, MSM and TG) in Nepal. This study included 43 studies conducted in different epidemic zones of Nepal involving 5900 FSW, 2637 MSM and TG, and 7105 PWID. Overall, behavioural interventions were found to be effective in terms of reducing HIV risk behaviours among FSW, MSM and TG, but not among PWID. Five studies identified through the literature search were excluded because the authors did not respond to our email or provide requested data. Nonetheless, given the inclusion of 43 large nationally representative surveys over a time period of 16 years, we argue that the risk of publication bias is small. It is thus reasonable to conclude that the pooled HIV prevalence rates for various key populations reported here reflect the true prevalence of HIV among key populations in Nepal. This assumption is supported by the alternative analysis performed using data from the AIDS Epidemic and Spectrum model.

A pooled analysis of HIV prevalence rates from studies conducted between 2004 and 2016 indicates an HIV prevalence of 11.9% among PWID, 5.0% among MSM and TG and 1.7% among FSW. These figures are considerably lower as compared to HIV prevalence rates from similar key populations in other Asian countries^26^.

Over eth last two decades, political and legal frameworks in Nepal have provided an enabling and supportive policy and structural environment that helped civil society and other actors combat this epidemic. Despite continuous challenges of being stigmatized because of belonging to a marginalized sub-population such as being a sex worker, gay, or living with HIV, the rapid response by the Nepal’s government to formally protect sexual and gender minorities from discrimination, violence and abuse, might have contributed to increased uptake of HIV prevention interventions by key populations. Furthermore, favourable changes in social capital (such as improved literacy and increased community participation) may have contributed to lower risk behaviors and vulnerability. Active engagement of civil society and community organizations in managing HIV related funding through a sector-wide approach and putting pressure on government and bilateral/multilateral agencies to resume closed or non-functioning HIV/AIDS-related programs have been crucial for Nepal’s success in reducing the incidence and prevalence of HIV^27^. Though needle exchange programs have been implemented in Nepal for many years, a persisting high HIV prevalence among PWID compared to other key populations, maybe contributed to the stigmatization associated with drug injection, leading to lower uptake of prevention services by PWID^28^. In addition, because of the open border between Nepal and India, cross-border movement for purchase of illicit drugs is still common, possibly undermining the impact of national prevention efforts^7^ or suggesting more cross-border collaboration.

Consistent condom use and safe injection practices are considered the most relevant behavioural indicators used to evaluate the effectiveness of HIV-preventive interventions among key populations. Our meta-analysis revealed that exposure to behavioural interventions in Nepal was associated with a significant increase in condom use in both vaginal and anal intercourse. However, this finding should be interpreted with caution keeping in mind that self-reported measures of condom use is sensitive to social desirability bias. In addition, there may be positive publication bias since positive accounts of program effectiveness are more likely to be reported and published.

Despite a significant proportion of respondents visiting HTS services, condom use remained below (61–73% in 2016) an optimal level and varied across key populations. Even though great strides have been made in increasing access to HIV testing facilities, a significantly lower proportion, across all key populations, visited facilities and the proportion was even lower for MSM and TG (for instance, in 2015, only 16.8% of MSM from Kathmandu Valley had visited a HTS facility in past 12 months compared to 23.5% of PWID, and 24.4% of FSW in same year) (Supplementary Table S2). The low uptake of HTS for MSM and PWID could be secondary to the continuous prejudice and discrimination people feel when accessing these services, especially when there is poor family support^29,30^. Additionally, further investigations are needed to understand why there is low uptake of HIV testing services despite provisions of services free of cost.

A relatively small proportion of individuals belonging to various key populations visit HIV testing services as compared to the numbers identified by peer educators (e.g., only 18.8% of MSM and TG visited HIV testing services in 2016 while 77.5% had participated in a peer-led intervention) (Supplementary Table S2). This gap should be carefully considered while evaluating HIV prevention interventions as several factors may interplay to cause high attrition or low uptake of formal HIV testing services such as ignorance, inadequate information in counselling, not finding it beneficial and worries about client privacy^1,31^. The first of UNAIDS’ 90-90-90 targets of 90% of all people being aware of their HIV status by 2020^32^ seems unattainable in Nepal unless the issue of discrimination and stigma is promptly taken into consideration.

Young people between the ages of 10–24 years, are constantly being recognized as a key population for HIV. There is an ongoing discussion around the world about whether a separate focus is required for young people and whether such programs should be integrated into on-going youth programs tailoring to the needs of young people^2^. Our review showed that for low-resource settings like Nepal, additional resources would be required to design separate programs for young people and the costs to develop new interventions will put additional strain on an already fragile health system.

The findings of this meta-analysis are consistent with prior meta-analyses showing positive impacts of interventions aimed to reduce risky behaviours such as poor condom use among FSW^33^ and MSM^13,34^. However, our analysis could not show that current interventions aimed to reduce unsafe injection among PWID were effective in Nepal, as opposed to the findings by Vlahov *et al*.^28^. Reviews have found that harm reduction programmes such as opioid substitution therapy and needle and syringe programmes are effective strategies in reducing injecting risk behaviour^35^. However, in Nepal, harm reduction programmes have very inadequate coverage and a low retention rate of 3% at six months in opioid substitution therapy and only 62 clean needles were exchanged per PWID per year in 2017 (much less than the World Health Organization recommendation of providing at least 200 needles and syringes per person per year)^36^. The explanation for the low uptake and poor coverage of harm reduction in Nepal needs further investigation using both quantitative and qualitative research since diverse group dynamics with different preferences, behaviours and circumstances, require tailored solutions, specifically adapted to the setting and population in need^37^. Furthermore, structural/contextual factors such as the criminalization of drug possession and use, societal discrimination against PWID and unrestricted cross-border (India-Nepal) movement for drug use might have impeded the success of previous interventions. Self-efficacy is often identified as important for predicting safer injection practices^38^, and new interventions would probably be more effective if they include components that strengthen the self-efficacy of PWID.

The major findings from this meta-analysis can be summarized in three main domains: (i) Overall the HIV prevalence among key populations in Nepal is low, but risky behaviors are persistent and further work is needed to reach the 90–90–90 targets; (ii) the reduction in HIV-related risk behaviors among FSW, MSM and TG can largely be attributed to behavioral interventions (peer education in formal and informal settings and HTS); and (iii) current interventions were effective regardless of age and epidemic zones among FSW, MSM and TG, but we could not find a positive impact of existing behavioural interventions among PWID.

While interpreting the findings, limitations of this analysis should be considered. Although we identified a large number of studies across these typologies, all were observational in design and relied on self-reported behavioural outcomes. Ideally, a randomized control trial design should be used to evaluate the effectiveness of interventions but none of the studies that used such a design met the inclusion criteria for this meta-analysis. However, it is often challenging to conduct randomized studies among hard to reach populations, and observational studies are still useful in such contexts, in particular related to stigmatized key populations.

Because of the cross-sectional nature of the studies included in this review, it is more difficult to draw firm conclusions about causality between an intervention and outcomes in terms of risk behaviours. Additionally, in most of the studies, the effectiveness of the intervention was assessed using self-reported risk behaviour which often is prone to social-desirability bias. However, in some individual studies, attempts were made to minimize this bias by using anonymous tools and not collecting any personal identifiers. Our meta-analysis revealed significant variation across studies (Q test and I^2^) which may be due to the small numbers of studies pooled into the meta-analysis and tests for heterogeneity is often influenced by low power^23^. A non-significant value of test of heterogeneity cannot rule out the presence of true heterogeneity between surveys without affecting the application of random effects model.

This meta-analysis provides strong evidence in support of behavioural interventions as promising strategies for addressing the HIV epidemic among FSW, MSM and TG, across age groups in Nepal and similar settings. However, the evidence of successful interventions for PWID, is more limited. A substantial gap exists between those reached by peer-driven interventions and those visiting HTS services; hence, efforts to optimize the continuum of HIV care can be improved by linking these interventions, while realizing that trust and reduction of stigma and discrimination are keys to increase the uptake of services by marginalized key populations. Further, contextual adaptation is the cornerstone for achieving maximum gain towards reducing the incidence of HIV.

## Supplementary information

Supplementary Information.

## Data Availability

The datasets used and/or analysed during the current study are available from the corresponding author on reasonable request.
