# Supplementary material for: Effectiveness of interventions for changing HIV related risk behaviours among key populations in low-income setting: A Meta-Analysis, 2001–2016
Source: Sci Rep. 2020 Feb 10;10:2197. doi: 10.1038/s41598-020-58767-0 (PMC7010789; doi:10.1038/s41598-020-58767-0)
Supplement: Supplementary file 1 — Supplementary Information. [file 41598_2020_58767_MOESM1_ESM.pdf]

Supplementary Information

Effectiveness of interventions for changing HIV related risk behaviours among key populations in low-income setting: A Meta-Analysis, 2001–2016

Keshab Deuba, Diksha Sapkota, Upendra Shrestha, Rachana Shrestha, Bir Bahadur Rawal, Komal Badal, Kathleen Baird, Anna Mia Ekström

Supplementary Table S1. Search strategy using Medical Subject Headings (MeSH) and keywords.

Search	Search terms
#1	'peer education' OR 'peer influence' OR 'peer support' OR 'peer support program' OR 'peer support intervnetions' OR 'peer services'
#2	'HIV intervention' OR HIV counseling OR 'HIV counseling and testing' OR voluntary testing
#3	Sex education OR (health edycation or health promotion) OR outreach programs OR outreach education OR HIV knowledge OR outreach centers OR outreach services OR drop in center OR drop in clinic OR drop in service
#4	1, 2, and 3 combined with 'OR'
#5	HIV knowledge OR safe sex practices OR risky behavior OR HIV attitudes OR HIV preventions OR health promotion
#6	Impact OR effect OR influence OR consequences
#7	5 and 6 combined with 'OR'
#8	Target population OR female sex workers OR (msm or men who have sex with men) OR (msm or homosexuals or gay) OR transgender OR injecting drug users
#9	Nepal OR nepalese OR nepali
#10	Combined 4, 7, 8, and 9 with OR
#11	Other examples of combination of search terms:<br>(("hiv"[MeSH Terms] OR "hiv"[All Fields]) AND ("prevention and control"[Subheading] OR ("prevention"[All Fields] AND "control"[All Fields]) OR "prevention and control"[All Fields])) OR (("hiv"[MeSH Terms] OR "hiv"[All Fields]) AND ("Intervention (Amstelveen)"[Journal] OR "intervention"[All Fields] OR "Interv Sch Clin"[Journal] OR "intervention"[All Fields]) AND effectiveness[All Fields]) AND ("nepal"[MeSH Terms] OR "nepal"[All Fields]) AND ("2001/01/01"[PDAT] : "2016/12/31"[PDAT])<br>VCT counseling AND HIV/effects AND Nepal[pl]<br>sexual health education/organization and administration AND Risk behaviors AND HIV<br>"drop in centre" OR "outreach centre" AND HIV<br>HIV Knowledge, attitude and practice AND behavior therapy/methods*<br>'human immunodeficiency virus' AND test* OR 'counseling' AND impact OR 'utilization' AND [2001-2016]/py AND 'nepal'<br>'behavioral approach' AND 'Human immunodeficiency virus'<br>'peer counseling'/exp OR 'peer counseling' AND ('human immunodeficiency virus'/exp OR 'human immunodeficiency virus')

Supplementary Table S2. Detailed descriptions of the included studies.

S.N.	Authors (reference number)	Year	Sample Size	Place of study	Intervention Description	Exposure measures (%)	Outcomes measures (%)	
**Female Sex Workers (FSWs)**							HIV prevalence	Condom use with most recent client
1	(51)	2016	610	22 Terai Highway districts	**Peer education and HTS**<br>a. Met or interacted with peer educators in last 12 month<br>b. DIC visit in last 12 month<br>c. HTS center visit in last 12 month	a. 73.4, b.71.1, c. 50.6	0.8 (0.3-2.0)	72.8
2	(52)	2016	342	Pokhara Valley		a. 38.9, b. 16.4, c.14.0	0.3	81.9
3	(53)	2015	500	Kathmandu Valley		a. 71.4, b. 54.6 c. 24.4	2.0 (0.37-3.63)	83.0
4	(54)	2012	410	22 Terai Highway districts		a. 47.0, b. 44.8, c.45.9	1.0 (0.3-1.8)	75.6
5	(55)	2011	593	Kathmandu Valley		a. 83.8, b. 33.7, c. 53.5	1.7 (0.7-2.7)	81.6
6	(56)	2011	345	Pokhara Valley		a. 78.6, b. 56.2, c.58.8	1.1 (0.0-2.3)	78.8
7	(57)	2009	600	22 Terai Highway districts		a. 87.2, b. 50.5, c. 65.2	2.3	84.8
8	(58)	2008	500	Kathmandu Valley		a. 59.6, b. 21.6, c. 32.8	2.2	75
9	(59)	2008	200	Pokhara Valley		a. 54.0, b. 25.5, c. 34.5	3	64.5
10	(60)	2006	500	Kathmandu Valley		a. 83.2, b. 31.2, c. 28.2	1.4	77.2
11	(61)	2006	200	Pokhara Valley		a. 50.0, b. 36.0, c. 15.0	2	75
12	(62)	2006	400	22 Terai Highway districts		a. 79.2%, b. 38.2%, c. 35.8%	1.5	66.30%
13	(63)*	2004	200	Pokhara Valley	No information	N/A	2	64.5
14	(64)*	2004	500	Kathmandu Valley	No information	N/A	2	74

**Men having sex with Men (MSM) and Transgender (TG)**							HIV prevalence	Condom use during last anal sex
15	(65)	2016	340	22 Terai Highway districts	**Peer education and HTS**<br>a. Met or interacted with peer educators in last 12 month<br>b. DIC visit in last 12 month<br>c. HTS center visit in last 12 month	a. 60.3, b. 56.2, c. 24.7	8.2 (4.1-15.7)	60.9
16	(66)	2015	400	Kathmandu Valley		a. 27.6, b 46.0. c. 16.8	2.4	80.9
17	(67)	2012	400	Kathmandu Valley		a. 71.3 , b. 55, c. 44.8	3.8 (2.0-6.0)	85.3
18	(68)	2009	400	Kathmandu Valley		a. 78.0, b. 54.5, c. 43.8	3.8	75.8
19	(69)	2007	400	Kathmandu Valley		a. 55.9, b. 30.7, c.9.6	3.3 (1.0-6.1)	71.6
20	(40)#	2013	339	15 districts of Nepal		85.5	N/A	22
21	(70)*	2004	358	Kathmandu Valley	HTS a. HTS center visit in last 12 month	N/A	3.9	66.7
**People who Inject Drugs (PWIDs)**							HIV prevalence	Unsafe injection in past week
22	(71)	2015	300	West to far west Terai districts		a.60.2 , b.69.0, c. 34.7	2.3	12.8
23	(72)	2015	340	Kathmandu Valley		a. 50.6, b. 63.5, c. 23.5	7.6	2.4
24	(73)$	2015	345	Pokhara Valley		a. 20.9, b. 28.4, c. 17.1	2.6	0.3
25	(74)	2015	360	Eastern terai districts		a. 43.3, b. 65.3, c. 22.8	8.3	8.6
26	(75)	2012	360	Eastern terai districts		a. 88.1, b. 96.1, c.49.7	8.1	8.1
27	(76)	2012	300	West to far west Terai districts		a.44.3, b. 65.0, c. 33.3	5.0	10.3

28	(77)$	2011	340	Kathmandu Valley		a. 60.6, b. 72.4, c.23.5	8.5	3.2
29	(78)	2011	345	Pokhara Valley		a. 85.5, b. 77.7, c. 35.4	4.6	2.3
30	(79)	2009	300	Kathmandu Valley		a. 81, b. 84.7, c. 24.3	20.0	8.0
31	(80)	2009	300	Pokhara Valley		a. 85.7, b. 93.7, c., 49.3	5.7	5.3
32	(81)	2009	345	Eastern terai districts		a. 90.7, b. 87.5, c. 33.6	8.1	10.7
33	(82)	2009	300	West to far west Terai districts		a.81.7, b. 77.3, c. 28.3	8.0	11.7
34	(83)	2007	300	Kathmandu Valley		a. 86.0, b. 76.0, c. 18.3	33.0	10.3
35	(84)	2007	300	Pokhara Valley		a. 67.3, b. 95.7, c. 44.7	8.7	8.3
36	(85)	2007	345	Eastern terai districts		a. 82.3, b. 80.0, c. 23.2	17.1	13.9
37	(86)	2007	300	West to far west Terai districts		a.80.3, b. 69.0, c. 14.0	11.0	10.3
38	(87)*	2005	300	Kathmandu Valley	HTS<br>a. HTS center visit in last 12 month	N/A	51.7	20.3
39	(88)*	2005	300	Pokhara Valley		N/A	21.7	14.7
40	(89)*	2005	339	Eastern terai districts		N/A	31.2	31.0
41	(90)*	2005	300	West to far west Terai districts		N/A	11.7	19
42	(91)*	2003	345	Pokhara Valley	No information	N/A	22.0	21.0
43	(92)*	2003	341	Eastern terai districts		N/A	35.5	48.1

Note: Peer education includes outreach education by peer educators and outreach educators and information, education, and counselling (IEC) activities conducted at drop-in centers

Outreach and peer educators provide education on STI, HIV and AIDS and preventive measures to key population. The teaching eas focused on HIV and STI transmission, condom use and demonstration. Drop-in centre: a safe place for key population to socialize and HIV related educational and counseling activities. HTS provides pre and post-test counseling, conduct HIV test and provide test results. Partner testing is recommended if diagnosed with STI and/or HIV

*Studies not included in meta-analyses assessing effectiveness of HIV interventions as no information about intervention

#Studies not included in calculating pooled prevalence.

@Studies not included in analysis as there is only one data point for the time.
$ Removed from the analysis of safe injecting behavior by outcome (met with OE/PE, visited DIC and HTC) due to insufficient responses to calculate odds

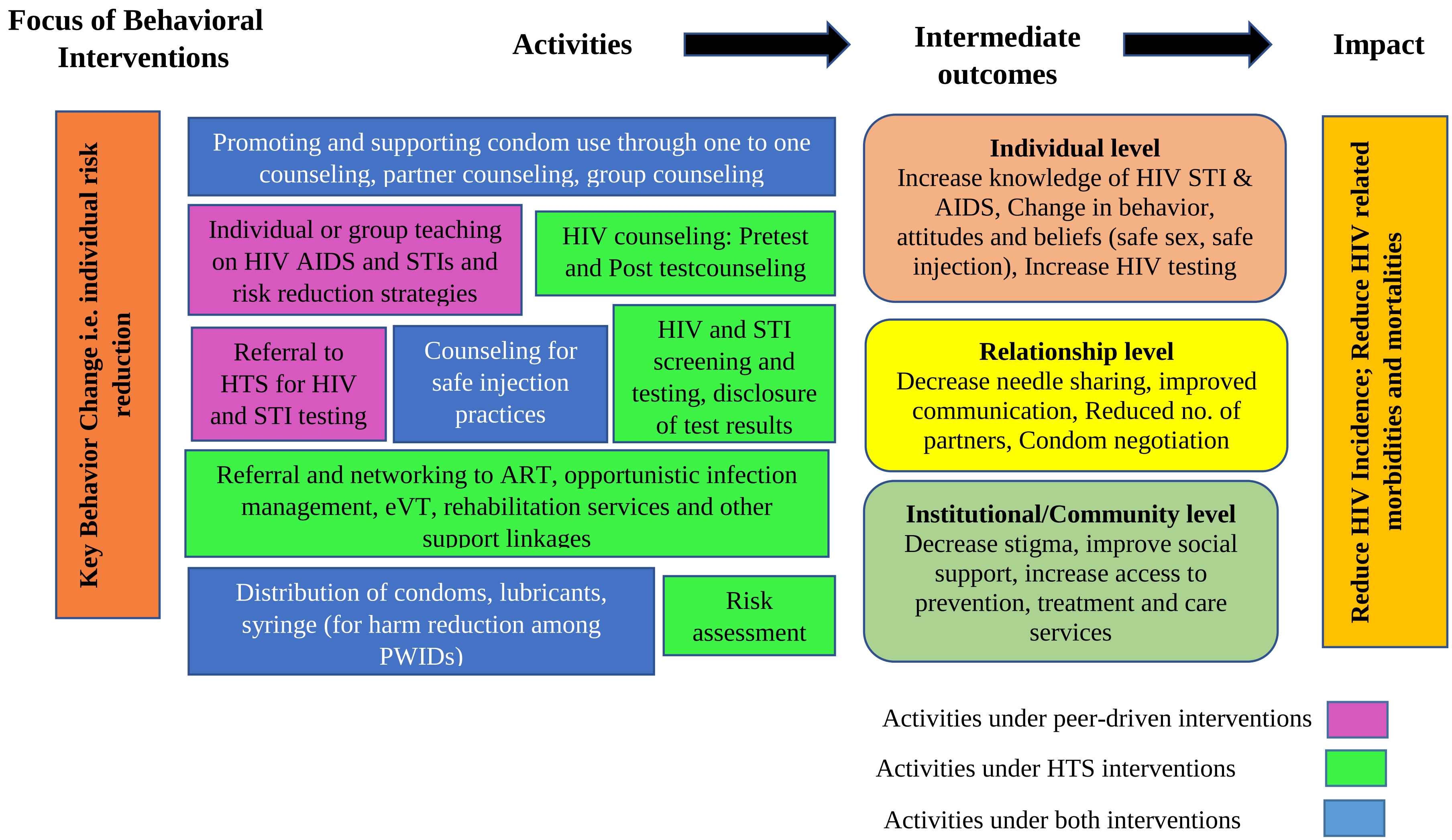

Supplementary Figure S1. Framework describing the process of how HIV related behavioral interventions works.

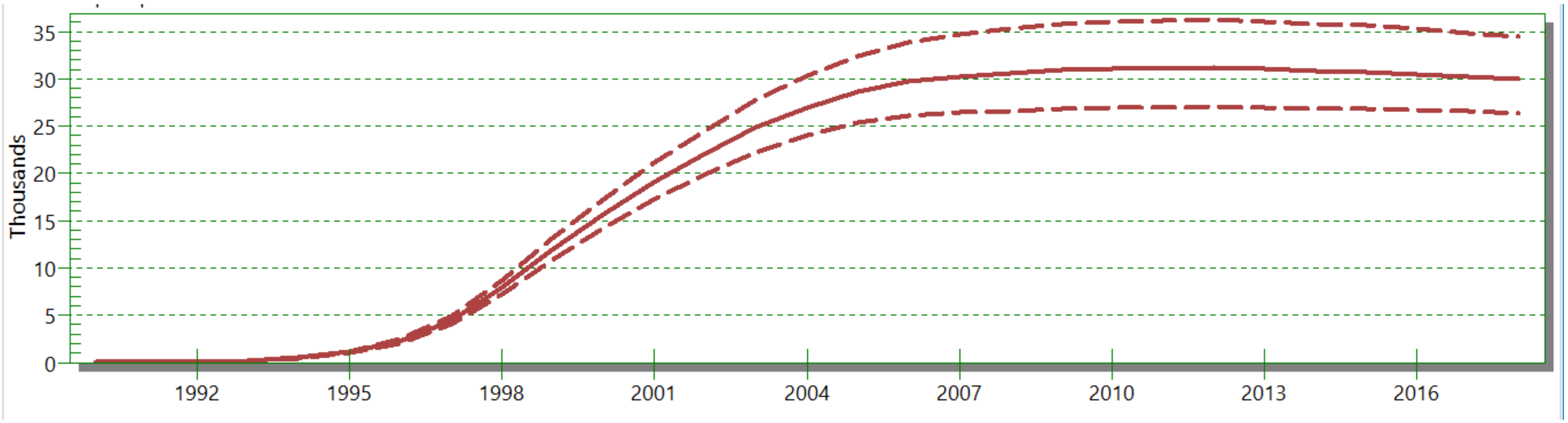

Supplementary Figure S2. HIV population in Nepal between 1990 and 2018.
